# Determinants and Clinical Impact of Visit-to-visit Blood Pressure Variability in Patients with Heart Failure with Preserved Ejection Fraction

**DOI:** 10.31662/jmaj.2024-0256

**Published:** 2025-06-27

**Authors:** Chinatsu Komiyama, Nobuyuki Kagiyama, Takuya Yuri, Akihiro Hayashida, Atsushi Hirohata, Kiyoshi Yoshida, Yuya Matsue, Tohru Minamino

**Affiliations:** 1 Department of Cardiovascular Biology and Medicine, Juntendo University Graduate School of Medicine, Tokyo, Japan; 2 Department of Cardiology, The Sakakibara Heart Institute of Okayama, Okayama, Japan

**Keywords:** heart failure preserved ejection fraction, hypertension, blood pressure variability

## Abstract

**Introduction:**

Blood pressure (BP) affects the prognosis of patients with heart failure with preserved ejection fraction (HFpEF). However, the implications of its variability are poorly understood. This study aimed to explore the determinants and prognostic significance of visit-to-visit BP variability (V2V-BPV) in HFpEF.

**Methods:**

Consecutive patients with HFpEF at the Sakakibara Heart Institute of Okayama underwent routine BP measurements. V2V-BPV, calculated as the coefficient of variation of systolic BP over one year, was assessed. The primary endpoint comprised all-cause mortality and heart failure hospitalization.

**Results:**

Among 288 outpatients with HFpEF (average age 73 ± 10 years, 60.8% male), BP was measured 6.1 ± 1.7 times, with a median V2V-BPV of 7.3%. The high V2V-BPV group (≥7.3%) had marginally but significantly elevated B-type natriuretic peptide (BNP) levels and higher Meta-Analysis Global Group In Chronic Heart Failure risk scores (MAGGIC scores). V2V-BPV was independently associated with an increased risk of the primary endpoints (hazard ratio 1.08 per percentage point; p = 0.025), even after adjustments for systolic BP, BNP, MAGGIC score, and the number of BP measurements. A similar relationship was observed between all-cause mortality and V2V-BPV (adjusted hazard ratio 1.12, p = 0.049 with MAGGIC score). Hemoglobin level was an independent predictor of high V2V-BPV in multiple sensitivity analyses.

**Conclusions:**

In patients with HFpEF, V2V-BPV was independently associated with adverse events, with hemoglobin level emerging as a determinant. Further research is warranted to determine whether BP stabilization can improve the prognosis of HFpEF.

## Introduction

The concept of heart failure has long been recognized, as evidenced by the Framingham criteria ^(1)^. This heterogeneous syndrome is generally classified based on cardiac function into heart failure with reduced ejection fraction (HFrEF) and heart failure with preserved ejection fraction (HFpEF), which was previously termed diastolic heart failure ^(2), (3)^. Studies have shown that HFpEF has a comparable prognosis to HFrEF, with a 1-year mortality of 22% ^(4)^. While many effective therapeutic options for HFrEF have been developed over the past 30 years ^(5)^, therapies for HFpEF remain a challenge, with only a few therapies proven to be effective ^(6), (7)^. Therefore, new therapeutic strategies for HFpEF are necessary.

Hypertension is one of the most common complications of HFpEF, and blood pressure (BP) control in patients with HFpEF is considered fundamental, as indicated by the class I recommendation in the major guidelines for heart failure ^(8)^. In addition to systolic and diastolic BP levels at a single point, recent studies have underscored the prognostic importance of BP variability. Studies have shown that BP variability is related to arterial stiffness ^(9), (10), (11)^, sympathetic nervous activity ^(12)^, and physical activity ^(13)^, and high BP variability may result in the progression of hypertensive organ damage, as represented by carotid intima-media thickness ^(14)^ and kidney injury such as albuminuria ^(15)^.

Despite the close association between hypertension and HFpEF, only 1 study from China has investigated BP variability and its implications in HFpEF. However, significant heterogeneity has been reported among different ethnic groups, even among Asian countries. We thought it would be important to verify such an association in a Japanese cohort. In addition, the underlying causes and mechanisms of this BP variability are unknown. Thus, the aim of this study was to investigate the determinants and prognostic impact of visit-to-visit BP variability (V2V-BPV) in patients with HFpEF.

## Materials and Methods

We retrospectively reviewed consecutive adult (≥20 years old) patients with HFpEF, whose BP was measured more than 4 times between October 2013 and September 2014 at the Sakakibara Heart Institute of Okayama. All patients were enrolled in October 2014, and the follow-up period started from this point. Follow-up data collection was completed by January 2018, and the dataset was finalized by November 2018. Subsequently, data analysis was conducted and completed in December 2023. The baseline data for all patients were defined as the most recent data within 3 months from October 2014. The diagnosis of heart failure was made by the attending physicians based on the Framingham criteria ^(1)^ before October 2013, and HFpEF was defined as left ventricular ejection fraction (LVEF) ≥50%. LVEF was assessed by transthoracic echocardiography performed by an experienced sonographer, and the biplane method of disks was employed in accordance with the published guidelines ^(16)^. Patients with chronic peritoneal dialysis or hemodialysis were excluded, as were those without LVEF measurements, and those hospitalized for cardiovascular diseases or who underwent intravenous diuretics between October 2013 and September 2014.

BP and pulse rate were measured twice by a nurse at the outpatient visit, and the mean values were used. BP measurement was primarily performed in a seated position, using the upper arm and an automated device. V2V-BPV was defined as the coefficient of variance of systolic BP measurements between October 2013 and September 2014. The number of BP measurements was defined as the total count of systolic BP recordings taken during the same period. Baseline characteristics, including age, sex, body mass index, comorbidities, medications, and laboratory data, were extracted from the electronic medical chart. The Meta-Analysis Global Group In Chronic Heart Failure risk score (MAGGIC scores) was calculated to assess the risk of each patient, as previously described, using age, gender, smoking habit, diabetes mellitus, chronic obstructive pulmonary disease, duration of heart failure, prescription of beta blockers and angiotensin-converting enzyme inhibitors/angiotensin receptor II blockers, LVEF, New York Heart Association (NYHA) class, serum creatinine, body mass index, and systolic BP. This risk score’s effectiveness in predicting all-cause mortality and/or heart failure hospitalization has been previously validated in Japanese patients ^(17)^.

The primary outcome was the composite of heart failure hospitalization and all-cause death. As a secondary outcome, we also investigated all-cause mortality. These outcomes were mainly tracked through the electronic medical chart. If more than 6 months had passed since the last outpatient visit, an additional telephone interview was conducted for follow-up.

To compare patients with high and low V2V-BPV, they were divided into 2 groups based on the median value of V2V-BPV, as no standard cutoff value had been established. Continuous variables were presented as mean ± standard deviation or median and interquartile range and were compared with Student’s *t*-test or Mann-Whitney U-test, depending on their distribution. Categorical variables were expressed as counts and percentages and were compared using the χ^2^ or Fisher’s exact test. To visualize the relationship between V2V-BPV and mean systolic BP, a scatterplot of these 2 variables was created, and Pearson’s correlation coefficient (r) was calculated.

Kaplan-Meier curves for the primary and secondary outcomes, stratified by V2V-BPV levels, were generated and compared using the log-rank test. Cox regression analysis was performed to explore the relationship between V2V-BPV and clinical outcomes. Multivariable-adjusted hazard ratios (HRs) and 95% confidence intervals (CIs) were calculated. We used the MAGGIC risk score and B-type natriuretic peptide (BNP) as covariates for the primary outcome, as the discrimination and calibration of the MAGGIC risk score have been well validated in Japanese patients with heart failure ^(18), (19)^, and adding BNP level at discharge has been shown to improve discrimination with adequate calibration ^(18)^. In addition, systolic BP and the number of BP measurements were used as covariates, as they might influence both V2V-BPV and the primary outcome; patients with unstable BP may have more frequent BP measurements and worse outcomes. For the model predicting all-cause death, due to the limited number of events, only 1 covariate was used in the Cox regression model to avoid the risk of overfitting.

To investigate the determinants of V2V-BPV, we constructed a multivariable logistic regression model to predict membership in the high V2V-BPV group using variables that differed significantly between the high and low V2V-BPV groups. In addition, we used multivariable linear regression models to predict V2V-BPV as a continuous variable, using variables that were significantly correlated with V2V-BPV values. Correlations with V2V-BPV were assessed using Pearson’s correlation coefficient (r), and stepwise variable selection using Akaike’s Information Criterion was employed.

All analyses were performed using R 4.0.3 (Vienna, Austria). All analyses were 2-sided. p-values < 0.05 were considered statistically significant.

We obtained written consent from all patients for the anonymization and publication of their information, including images.

## Results

**Figure 1.**
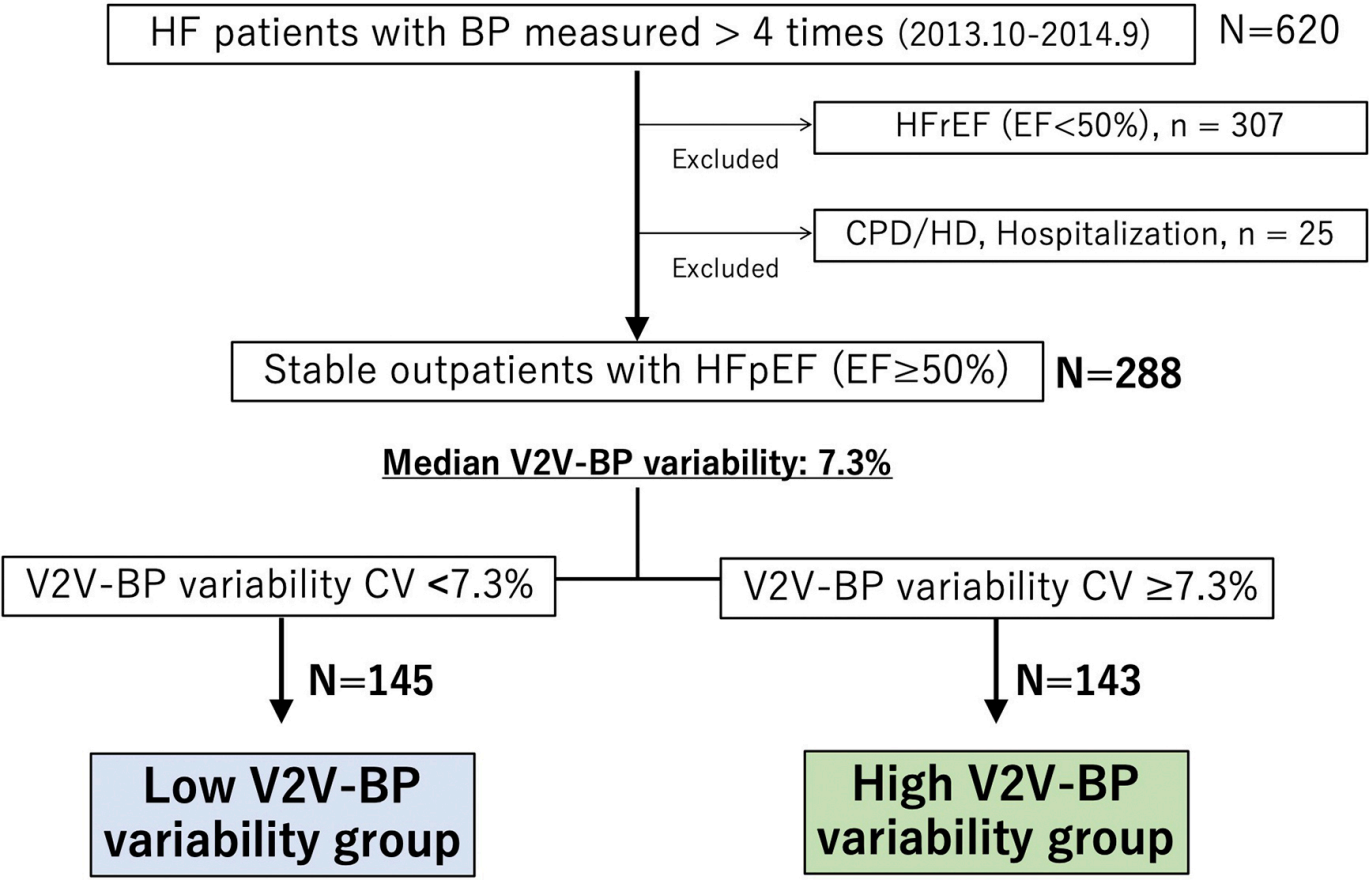
The patient enrollment flowchart. Among 620 patients with heart failure who measured BP more than 4 times during the study period, 307 were excluded due to reduced LVEF, and an additional 25 were excluded based on other aforementioned criteria. As a result, 288 stable outpatients with HFpEF were included in the analysis. Patients were divided into 2 groups based on the median V2V-BPV value (7.3%). BP: blood pressure; HFpEF: heart failure with preserved ejection fraction; LVEF: left ventricular ejection fraction; V2V-BPV: visit-to-visit BP variability.

**Figure 2.**
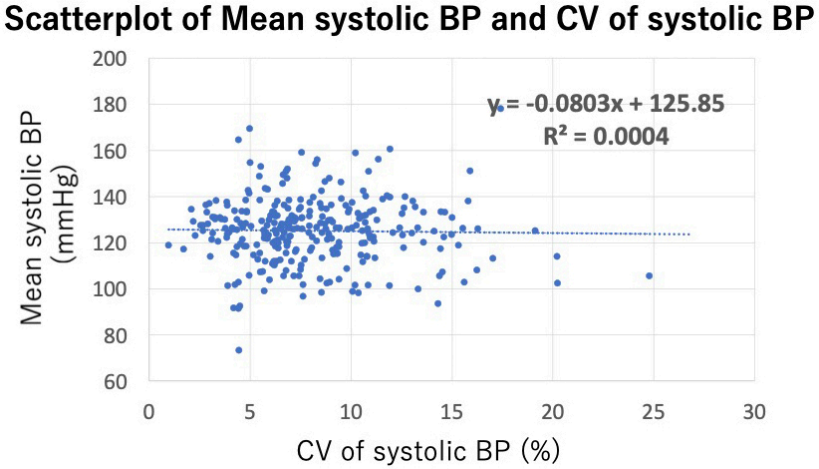
The scatterplot of V2V-BPV and Mean systolic BP. There was no significant association between V2V-BPV and Mean systolic BP (r = −0.009, p = 0.89). x indicates the CV of systolic BP, and y indicates mean systolic BP. The scatterplot was created using Microsoft Excel (Microsoft Corp., Redmond, WA, USA). BP: blood pressure; CV: Coefficient of Variation; V2V-BPV: visit-to-visit BP variability.

Among 620 patients with heart failure who measured BP more than 4 times during the study period, 307 were excluded due to reduced LVEF, and an additional 25 were excluded based on other aforementioned criteria. As a result, 288 stable outpatients with HFpEF were included in the analysis. The patient enrollment flowchart is shown in Figure 1. The average number of hospital visits per year was 6.1 ± 1.7, and the median V2V-BPV was 7.3% (1.0%-24.8%). Among the 288 participants, only 4 cases (1.4%) had BP measurements taken exclusively in either summer or winter, while the remaining 284 cases (98.6%) had measurements taken across both summer and winter. Patients were divided into 2 groups based on this median V2V-BPV value. The scatterplot of V2V-BPV and systolic BP is presented in Figure 2, showing no significant association between V2V-BPV and systolic BP (r = −0.009, p = 0.89).

Baseline characteristics of the study participants are presented in Table 1. There were no significant differences in age, gender, medication, or comorbidities. Physical examination showed no significant differences in systolic and diastolic BP or pulse rate. Laboratory tests revealed that creatinine and BNP levels in the high V2V-BPV group were significantly higher than those in the low V2V-BPV group, and hemoglobin levels were significantly lower in the high V2V-BPV group. LVEF was not significantly different between the 2 groups. The MAGGIC score indicated a significantly higher risk in the high V2V-BPV group.

**Table 1.** Patient Characteristics.

Patient characteristics	All N = 288	Low V2V-BPV group n = 145	High V2V-BPV group n = 143	p-Value	n (%) Missing
Age, years old	73 ± 10	72 ± 11	74 ± 10	*0.050*	*0 (0%)*
Female, n (%)	113 (39.2%)	54 (37.2%)	59 (41.3%)	*0.546*	*0 (0%)*
BMI, kg/m^2^	23.6 ± 3.9	24.0 ± 4.0	23.1 ± 3.7	*0.071*	*0 (0%)*
BSA, m^2^	1.61 ± 0.20	1.64 ± 0.2	1.59 ± 0.21	*0.020*	*0 (0%)*
Systolic BP, mmHg	125 ± 14	125 ± 14	125 ± 15	*0.822*	*0 (0%)*
Diastolic BP, mmHg	69 ± 12	70 ± 11	68 ± 12	*0.140*	*0 (0%)*
Heart rate/min	70 ± 13	70 ± 13	71 ± 13	*0.283*	*0 (0%)*
V2V-BPV	8.1 ± 3.6	5.3 ± 1.4	10.9 ± 3.0	*<0.001*	*0 (0%)*
Number of BP measurements	6.2 ± 1.7	6.1 ± 1.8	6.2 ± 1.7	*0.561*	*0 (0%)*
LV ejection fraction, %	63 ± 7	63 ± 7	64 ± 6	*0.266*	*0 (0%)*
**Medications, n (%)**
ACEi/ARB	161 (55.9%)	81 (55.9%)	80 (55.9%)	*>0.999*	*0 (0%)*
β blockers	160 (55.6%)	80 (55.2%)	80 (55.9%)	*0.906*	*0 (0%)*
MRA	52 (18.1%)	26 (17.9%)	26 (18.2%)	*>0.999*	*0 (0%)*
Statin	160 (55.6%)	86 (59.3%)	74 (51.7%)	*0.236*	*0 (0%)*
Calcium channel blockers	134 (46.5%)	62 (42.8%)	72 (50.3%)	*0.237*	*0 (0%)*
Diuretics	110 (38.2%)	48 (33.1%)	62 (43.4%)	*0.070*	*0 (0%)*
**Co-morbidities, n (%)**
Hypertension	151 (52.4%)	72 (49.7%)	79 (55.2%)	*0.348*	*0 (0%)*
Atrial fibrillation	149 (51.7%)	73 (50.3%)	76 (53.1%)	*0.639*	*0 (0%)*
Coronary artery disease	124 (43.1%)	59 (40.7%)	65 (45.5%)	*0.475*	*0 (0%)*
Chronic obstructive pulmonary disease	27 (9.4%)	16 (11.0%)	11 (7.7%)	*0.420*	*0 (0%)*
Diabetes mellitus	154 (53.5%)	75 (51.7%)	79 (55.2%)	*0.557*	*0 (0%)*
Current smoker	90 (31.3%)	44 (30.3%)	46 (32.2%)	*0.800*	*0 (0%)*
History of heart failure hospitalization	89 (30.9%)	41 (28.3%)	48 (33.6%)	*0.373*	*0 (0%)*
NYHA				*0.105*	
I	139 (48.3%)	77 (53.1%)	62 (43.4%)
II	108 (37.5%)	53 (36.6%)	55 (38.5%)
III	41 (14.2%)	15 (10.3%)	26 (18.2%)
**Laboratory test**
Albumin, g/dL	4.12 ± 0.33	4.16 ± 0.3	4.08 ± 0.35	*0.135*	*97*
					*(33.7%)*
BUN, mg/dL	20 ± 11	19 ± 13	21 ± 8	*0.167*	*0 (0%)*
Creatinine, mg/dL	0.97 [0.83 - 1.18]	0.93 [0.82 - 1.13]	1.01 [0.86 - 1.27]	*0.037*	*0 (0%)*
eGFR, mL/min/1.73 m^2^	44 ± 16	45 ± 14	42 ± 17	*0.106*	*0 (0%)*
Sodium, mEq/L	140 ± 2.8	140 ± 2.9	141 ± 2.7	*0.596*	*4 (1.4%)*
HbA1c, %	6.40 ± 0.84	6.36 ± 0.87	6.43 ± 0.81	*0.527*	*16 (5.6%)*
T-Cho, mg/dL	171 ± 35	171 ± 30	171 ± 40	*0.873*	*14 (4.9%)*
Hemoglobin, g/dL	12.81 ± 1.81	13.21 ± 1.64	12.41 ± 1.88	*<0.001*	*0 (0%)*
BNP, pg/mL	72 [34 - 157]	61 [28 - 143]	90 [38 - 216]	*0.030*	*0 (0%)*
**MAGGIC score (points)**	22 ± 7	21 ± 7	23 ± 7	*0.004*	*0 (0%)*

BMI: body mass index; BNP: brain natriuretic peptide; BP: blood pressure; BSA: body surface area; BUN: blood urea nitrogen; eGFR: estimated glomerular filtration rate; LV: left ventricular; MAGGIC: Meta-Analysis Global Group in Chronic heart failure; NYHA: New York Heart Association; T-Cho: total cholesterol; V2V-BPV: visit-to-visit BP variability.

**Figure 3.**
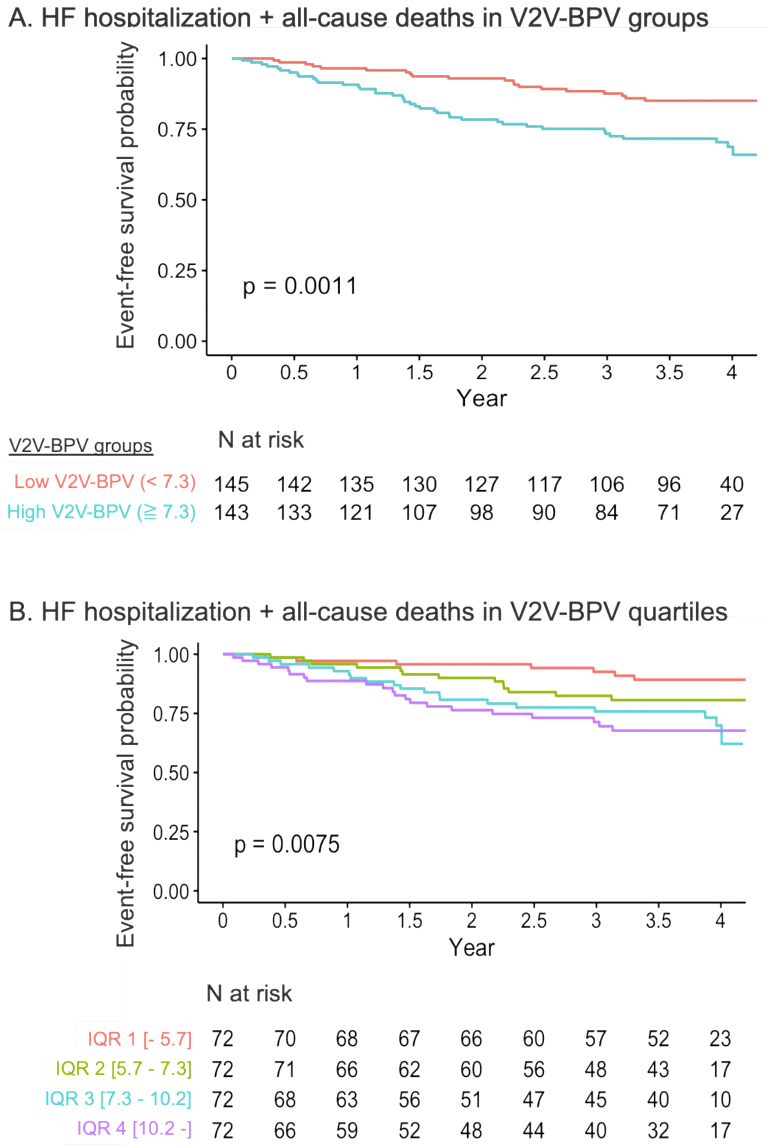
Kaplan-Meier curve analysis for the primary endpoint of a composite of all-cause mortality and heart failure hospitalization It showed that the high V2V-BPV group had a significantly higher event rate for the primary outcomes than the low V2V-BPV group (p = 0.001; Figure A). When stratified by the quartile of V2V-BPV, the curves indicated that higher V2V-BPV was associated with a higher event rate (p = 0.008, Figure B). BP: blood pressure; V2V-BPV: visit-to-visit BP variability.

During the median follow-up of 1434 (1093-1623) days, 60 patients (20.8%) experienced the primary endpoints, and 23 (8.0%) died. Kaplan-Meier curve analysis showed that the high V2V-BPV group had a significantly higher event rate for the primary outcomes than the low V2V-BPV group (p = 0.001; Figure 3A). When stratified by the quartile of V2V-BPV, the curves indicated that higher V2V-BPV was associated with a higher event rate (p = 0.008; Figure 3B). Cox proportional hazards models revealed that V2V-BPV level was independently associated with the primary outcomes after adjustment for MAGGIC score, BNP level, mean systolic BP, and the number of BP measurements (HR 1.08, 95% CI 1.01-1.16 per %, p = 0.025).

**Figure 4.**
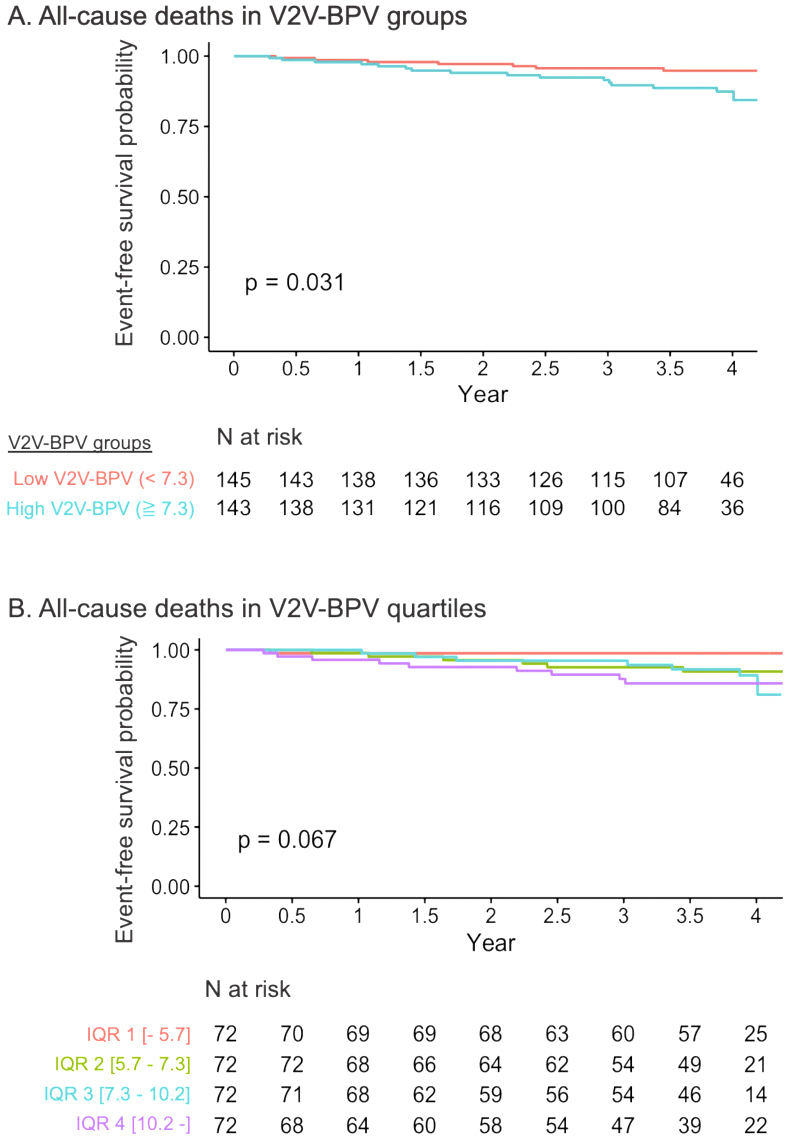
Kaplan-Meier curve analysis for the secondary endpoint of all-cause death The high and low V2V-BPV groups divided by the median value of V2V-BPV showed significantly different event risks. The quartile groups of V2V-BPV revealed a numerically higher risk in the higher V2V-BPV group, although the difference was not statistically significant, possibly due to the small number of events. BP: blood pressure; V2V-BPV: visit-to-visit BP variability.

For the secondary endpoint of all-cause death, the high and low V2V-BPV groups divided by the median value of V2V-BPV, showed significantly different event risks. The quartile groups of V2V-BPV revealed a numerically higher risk in the higher V2V-BPV group, although the difference was not statistically significant, possibly due to the small number of events (Figure 4A and B). Given the limited number of events (number of events = 23), the Cox proportional hazard model for predicting all-cause death was constructed with only 2 variables to avoid the risk of overfitting. V2V-BPV was also significantly associated with all-cause death after adjustment for either MAGGIC score (HR 1.12, 95% CI 1.00-1.25 per %, p = 0.049) or BNP levels (HR 3.89, 95% CI 1.38-10.94, p = 0.011) (Table 2).

**Table 2.** HRs of V2V-BPV for the Primary and Secondary Outcome.

A. All-cause deaths and heart failure hospitalization			
Multivariable Cox regression model	HR	95% CI	p-Value
V2V-BPV, per %	1.08	1.01-1.16	*0.025*
MAGGIC score, per score	1.17	1.12-1.22	*<0.001*
Mean systolic BP, per mmHg	1.01	0.99-1.03	*0.210*
BNP, per 10 ng/mL	1.02	1.00-1.03	*0.015*
Number of BP measurements, per time	1.01	0.86-1.18	*0.920*
**B. All-cause deaths**
**Multivariable Cox regression model1**	**HR**	**95% CI**	**p-Value**
V2V-BPV, per %	1.120	1.00-1.25	*0.049*
MAGGIC score, per score	1.290	1.20-1.38	*<0.001*
**Multivariable Cox regression model2**	**HR**	**95% CI**	**p-Value**
V2V-BPV, per %	1.150	1.03-1.27	*0.011*
BNP, per 10 ng/mL	1.040	1.02-1.05	*<0.001*

BMI: body mass index; BNP: brain natriuretic peptide; BP: blood pressure; BSA: body surface area; BUN: blood urea nitrogen; CI: confidence interval; eGFR: estimated glomerular filtration rate; HR: hazard ratio; LV: left ventricular; MAGGIC: Meta-Analysis Global Group in Chronic heart failure; NYHA: New York Heart Association; T-Cho: total cholesterol; V2V-BPV: visit-to-visit BP variability.

To investigate the determinants of high V2V-BPV, a multivariable logistic regression model was constructed using variables that were significantly different between the 2 groups. The only baseline characteristic independently associated with high V2V-BPV was the serum hemoglobin level (Table 3A). Additionally, linear regression analysis was performed to predict the continuous value of V2V-BPV using variables that had shown a significant correlation with V2V-BPV. The Pearson correlation coefficient (r) was significant for the MAGGIC score (r = 0.19, p = 0.001), hemoglobin level (r = −0.17, p = 0.004), BNP (r = 0.16, p = 0.006), serum albumin level (r = −0.19, p = 0.008), age (r = 0.13, p = 0.028), and NYHA class (r = 0.13, p = 0.032). The linear regression model with stepwise variable selection indicated that hemoglobin and albumin levels were significantly associated with higher V2V-BPV (Table 3B).

**Table 3.** Determinants of V2V-BPV.

A. Determinants of dichotomized high V2V-BPV			
Multivariable logistic regression model	HR	95% CI	p-Value
Hemoglobin, per g/dL	0.814	0.688 - 0.963	0.015
BNP, per 10 ng/mL	1.013	0.989 - 1.037	0.290
MAGGIC score, per score	1.035	0.965 - 1.110	0.330
Creatinine, per mg/dL	0.849	0.525 - 1.373	0.500
Age, per year	0.875	0.585 - 1.311	0.510

**B. Determinants of V2V-BPV as a continuous variable**
**Multivariable linear regression model1**	**Beta**	**95% CI**	**p-Value**
Hemoglobin, per g/dL	-0.29	-0.57 - -0.01	0.040
Albumin, per mg/dL	-1.52	-3.04 - -0.01	0.046
BNP, per 10 ng/mL	0.03	-0.01 - 0.06	0.140

BNP: brain natriuretic peptide; CI: clinical interval; HR: hazard ratio; MAGGIC: Meta-Analysis Global Group in Chronic heart failure: V2V-BPV; visit-to-visit BP variability.

## Discussion

In this retrospective analysis, we found that (1) patients with high V2V-BPV had lower body mass and serum hemoglobin levels, as well as higher BNP and MAGGIC scores than those with low V2V-BPV; (2) higher V2V-BPV was significantly associated with the composite outcome of heart failure hospitalization and all-cause deaths, as well as with all-cause death itself; and (3) hemoglobin level was an independent determinant of V2V-BPV. These findings emphasize the importance of BP stability in patients with HFpEF and pave the way for new research into BP stabilization as a potential treatment avenue for this challenging disease.

**Figure 5.**
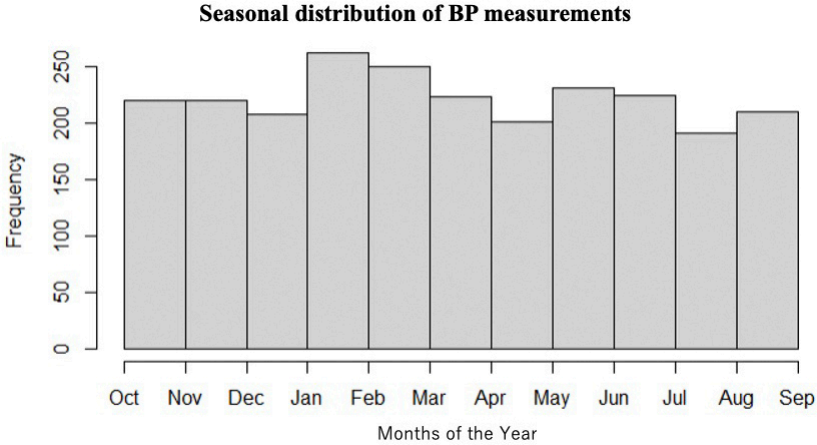
Seasonal distribution of BP measurements. BP measurements were evenly distributed in every season. BP: blood pressure.

BP is characterized by marked fluctuations over both short- and long-term periods. Types of BP variability include beat-to-beat variations related to breathing and the autonomic nervous system; within-day BP variability (minute-to-minute, hour-to-hour, or day-to-night) related to physical activity and sleep over 24 hours; day-to-day BP variability within a several-day period; V2V-BPV between outpatient visits; seasonal variability; and changes over time due to aging. In this study, BP measurements were performed >6 times on average, and their timing was distributed evenly across all seasons, as shown in Figure 5. Since most of the patients underwent BP measurements throughout the year, we believe that the results of this study reflect V2V-BPV considering seasonal variation, and it would be difficult to separate the influence of seasonal variation.

Among these, the association of V2V-BPV with the development, progression, and severity of cardiac, vascular, and renal damage, as well as with an increased risk of cardiovascular events and mortality, has been widely recognized ^(20), (21), (22)^. Post-hoc analyses of large intervention trials in patients with hypertension have shown that intraindividual and interindividual V2V-BPVs were strong predictors of cardiovascular morbidity and mortality ^(23)^. Hata et al. ^(24)^ reported that V2V-BPV was an independent risk factor for macrovascular diseases (coronary heart disease and stroke) in patients with type 2 diabetes mellitus. Okada et al. ^(25)^ reported that V2V-BPV was a marker of cardiac diastolic function. Nwabuo et al. ^(14)^ reported that V2V-BPV in early adulthood is associated with increased carotid intima-media thickness. Wang et al. ^(15)^ observed that visit-to-visit BP variability is associated with kidney injury such as albuminuria.

Although studies have linked HFpEF with the above-mentioned factors related to V2V-BPV, including diastolic function, atherosclerosis, and renal function ^(26)^, few studies have investigated the direct association between HFpEF and V2V-BPV. Zhang et al. ^(27)^ studied the prognostic value of V2V-BPV in patients with HFpEF enrolled in the TOPCAT study. They showed that cardiovascular death and heart failure hospitalization, as well as all-cause deaths, were associated with V2V-BPV independently of mean systolic BP. The cohort in this study was, on average, 6 years older (73 years old vs. 63 years), had a lower proportion of (NYHA) III/IV heart failure (14% vs. 29%), and a lower estimated glomerular filtration rate (44.0 ml/min/1.73 m^2^ vs. 68.9 ml/min/1.73 m^2^). The same association between V2V-BPV and clinical outcome was confirmed in this Asian cohort.

In addition to the aforementioned study, our research investigated the determinants of high V2V-BPV and revealed that a lower hemoglobin level is significantly associated with high V2V-BPV across multiple analyses. Regarding the broader context of the association between hemoglobin level and BP variability, previous studies have indicated a correlation between hemoglobin levels, BP, and hypertension. Some have demonstrated a positive association between hemoglobin levels and both systolic and diastolic BP in individuals with or without hypertension ^(28), (29), (30), (31)^, while other studies found that anemia was associated with nocturnal hypertension ^(32), (33)^. Both anemia and nocturnal hypertension are well-known factors associated with increased cardiovascular risks, especially in patients with HFpEF ^(34), (35), (36)^. The association between anemia and BP variability can be attributed to three mechanisms. First, anemia impairs the ability of red blood cells (RBCs) to produce nitric oxide (NO), an essential regulator of vasodilation. RBCs synthesize NO via nitrite reduction under hypoxic conditions and through endothelial nitric oxide synthase (eNOS) activation under normoxic conditions. Anemia reduces the NO bioavailability from RBCs, thereby compromising vascular regulation. Second, vascular dysfunction has been implicated, as anemia depletes the circulating NO pool, leading to instability in vascular tone and increased BP fluctuations ^(37), (38)^. In response to this NO depletion, vascular eNOS expression and activity are upregulated, potentially overstimulating BP regulatory mechanisms and amplifying variability. Third, anemia-induced hypoxia activates the sympathetic nervous system, which attempts to stabilize BP through increased heart rate and vasoconstriction. Furthermore, reduced renal perfusion stimulates the renin-angiotensin-aldosterone system, resulting in sodium retention and fluid volume expansion, which also contribute to BP variability. These mechanisms may play important roles in the present results of the association between high V2V-BPV and anemia, although the underlying mechanisms are unknown and further research is needed.

In the current study, systolic and diastolic BP in both groups were well controlled, averaging around 125/70 mmHg. Our findings highlighted that even in patients with well-controlled BP, V2V-BPV may have additional prognostic importance and should be monitored in patients with HFpEF. Further studies are anticipated to investigate the effectiveness of treatment strategies to reduce BP variability in this patient population. Although numerous classes of antihypertensive drugs have proven effective in clinical practice, none have been established specifically to stabilize BP variability. The ASCOT trial demonstrated that amlodipine reduced BP variability more than atenolol ^(39)^. Other studies have also confirmed the beneficial effect of calcium-channel blockers and diuretics in reducing BP variability compared with other antihypertensive agents ^(40), (41), (42), (43)^. Yet, there is a lack of randomized trials to elucidate the abilities of different types of drugs to stabilize BP variability, and the present study underscores the need for future research in this area.

Our study must be interpreted within the context of its limitations. First, this was a retrospective study conducted at a single Japanese institute and included a relatively small number of patients. Further studies in different settings are warranted to confirm these results. Second, we did not use ambulatory BP monitoring to capture short-term BP variability and nocturnal hypertension, which might influence the results as confounders. Third, the intervals between outpatient visits were not standardized. Although we confirmed that the results were significant even after adjusting for the number of BP measurements, prospective studies with routine visits and a standardized protocol are needed. Although the method for BP measurement was consistent for most patients, as it followed our routine clinical procedure, some patients may have undergone a different measurement method (e.g., in a supine position or using a manual device), which was not captured due to the retrospective nature of the study. The measurement method used in our study aligns with the definition of “office blood pressure” according to the Japanese Society of Hypertension Guidelines 2019 ^(44)^. Previous studies have suggested that automated BP measurements conducted by non-physician staff can be reliable and accurate ^(45), (46)^. While we think that these measurements can be considered as office BP in our study, we understand that this is still open to discussion. Fourth, this dataset did not include details of changes in medication between October 2013 and September 2014, which might impact V2V-BPV. However, drug adjustments might not have been frequent, as the majority of the patients in this study were stable heart failure patients who did not require hospitalization and treatment during this period, and their BP was well controlled on average. Fifth, the study was conducted between 2013 and 2014, and it should be acknowledged that drug treatment might differ from the current standard.

### Conclusions

Patients with high V2V-BPV had significantly higher rates of all-cause mortality and heart failure hospitalization than those with low V2V-BPV, even in a population with well-controlled BP levels. Hemoglobin appeared to be an independent predictor of V2V-BPV. Future studies are warranted to test whether the stabilization of BP can improve outcomes in patients with HFpEF.

## Article Information

### Conflicts of Interest

Nobuyuki Kagiyama is affiliated with a department endowed by grants from Paramount Bed Co., Ltd., received research grants from EchoNous. Inc. and AMI Inc., and received an honorarium from Novartis Japan, Otsuka Pharma, Eli Lilly, and Nippon Boehringer Ingelheim outside the submitted work. Yuya Matsue received an honorarium from Otsuka Pharmaceutical Co., Novartis Japan, AstraZeneca K.K., Ono Pharmaceutical Co., Ltd., Kyowa Kirin Co., Ltd., Bayer Japan, and Pfizer, Inc., and research funding outside the submitted work from Nippon Boehringer Ingelheim Co., Ltd., Pfizer Inc., Otsuka Pharmaceutical Co., EN Otsuka Pharmaceutical Co., Ltd., and Roche Diagnostics Japan. The other authors have no conflicts of interest to declare.

### Author Contributions

Conception or design of the work: Nobuyuki Kagiyama, Yuya Matsue, Tohru Minamino. Data acquisition: All authors. Analysis: Takuya Yuri, Akihiro Hayashida, Atsushi Hirohata, Kiyoshi Yoshida. Interpretation of data for the work: All authors. Drafting the work: Chinatsu Komiyama, Nobuyuki Kagiyama, Takuya Yuri. Reviewing the work critically for important intellectual content: Akihiro Hayashida, Atsushi Hirohata, Kiyoshi Yoshida, Yuya Matsue, Tohru Minamino. Final approval of the version to be published: All authors. Agreement to be accountable for all aspects of the work in ensuring that questions related to the accuracy or integrity of any part of the work are appropriately investigated and resolved: All authors.

### Approval by Institutional Review Board (IRB)

IRB Approval Code: B201711-01

Name of the Institution: The Sakakibara Heart Institute of Okayama
